# Awareness, acceptability and uptake of cervical cancer vaccination services among female secondary school teachers in Enugu, Nigeria: a cross-sectional study

**DOI:** 10.11604/pamj.2021.39.62.28824

**Published:** 2021-05-21

**Authors:** Joseph Tochukwu Enebe, Nympha Onyinye Enebe, Chuka Chike Agunwa, Obinna Chukwuebuka Nduagubam, Innocent Igwebeze Okafor, Elias Chike Aniwada, Emmanuel Nwabueze Aguwa

**Affiliations:** 1 Department of Obstetrics and Gynaecology, Enugu State University of Science and Technology College of Medicine/Teaching Hospital, Parklane, Enugu, Nigeria,; 2 Department of Community Medicine, University of Nigeria Teaching Hospital (UNTH), Enugu, Nigeria,; 3 Department of Paediatrics, Enugu State University of Science and Technology College of Medicine/Teaching Hospital, Parklane, Enugu, Nigeria

**Keywords:** Cervical cancer, human papillomavirus, vaccination, teachers

## Abstract

**Introduction:**

cervical cancer is a major cause of morbidity and mortality in women and vaccination of adolescents with human papilloma virus (HPV) vaccines is a major preventive strategy for this cancer. Despite the usefulness of cervical cancer vaccines, significant gaps still exist in the level of awareness and acceptability of the vaccine among women. This study aimed to determine the level of awareness, acceptability, and identify the factors associated with the uptake of this vaccine by female secondary school teachers in Enugu, Nigeria.

**Methods:**

a cross-sectional study of 377 female teachers in Enugu metropolis was undertaken between July and October 2017. A structured interviewer-administered pretested questionnaire was used for data collection and SPSS used for analysis.

**Results:**

less than half (41.9%) of the respondents had good knowledge of the cervical cancer vaccine. The majority of the respondents (93.6%) accepted the vaccine and will recommend it for their children and students. Only 14.6% and 9.0% of the respondents have taught their students about cervical cancer or HPV vaccines and had a programme in their school that addresses cervical cancer or cervical cancer vaccination respectively. Only 3.4% of the respondents have been vaccinated while 5.6% of their children or relatives have received the HPV vaccine. Previous vaccination of participants (p = 0.000), existing programmes addressing cervical cancer in schools of respondents (p = 0.000), participants having taught students about cervical cancer (p = 0.025) and high economic status (p = 0.013) significantly increased the likelihood of participants vaccinating their adolescent daughters/relatives. Extremes of age (p = 0.001) and being the head of their families (p = 0.002) significantly reduced the likelihood of the daughters/relatives to be vaccinated. Only previous history of vaccination of the respondents predicted the vaccination of their children and relatives (AOR = 6.069; 95% CI; < 0.0001-0.041).

**Conclusion:**

the overall knowledge of the HPV vaccine was low but the acceptability was high among respondents who were aware of the vaccines. Vaccine uptake among children/family members of the respondents was low. The introduction of cervical cancer vaccination education of the teachers in the secondary schools will help improve cervical cancer vaccination and the uptake among adolescent´s populations in the country.

## Introduction

Cervical cancer is the second most common cause of death among women globally and the commonest genital tract cancer especially among women residing in developing countries [1]. This disease is almost eliminated among women in the developed world due to the high use of different cervical cancer screening methods available via well-mapped screening programmes for cervical cancer [2]. However, in developing countries such as Nigeria; the diagnosis of cervical cancer is almost a death sentence with a huge economic significance which ordinarily would be reduced if the use of cervical cancer vaccines is increased in our environment [3]. Nigeria's population is about 1% of the world population but the country surprisingly contributes 10% of the global cervical cancer burden and habours the heaviest cervical cancer burden in Africa. In Nigeria, cervical cancer is the commonest genital cancer in women and it accounts for the death of about 8000 women annually and this gives an average of death from cancer per hour [4].

The discovery of human papillomavirus (HPV) as the agent that causes cervical cancer brought some hope on how cervical cancer can be prevented and treated. While developed countries have well established cervical cancer screening guidelines and have reduced the incidence of cancer in those countries considerably, the developing countries have no guidelines for screening and the burden of cervical cancer remains high in them. In addition, the discovery of the cervical cancer vaccine has further increased the hope of eradication of this virus globally. The high cost of living and poverty in developing countries has reduced the uptake of these cervical cancer vaccines. The non-existence of policy on the provision of this vaccine by the government to protect women and even men from HPV related cancers in Nigeria has been worsened the different levels of awareness and acceptability of this vaccine by different populations. Health education through the use of radio programmes, community outreach, conferences, and other forms of social marketing targeted towards women and parents of children that are likely to benefit from HPV vaccination, therefore, becomes very necessary and remains a viable option. This health education can be given at different levels by different groups of people such as teachers who can impact health education on others. Therefore, understanding the existing levels of knowledge of cervical cancer and cervical cancer vaccine among different populations that have frequent contact with parents of children or children that need this vaccination must be encouraged at all levels. The awareness of cervical cancer and cervical cancer vaccines among the women groups varies according to the profession and background of the women [5]. While studies [6, 7] have demonstrated a high level of awareness among female health workers; the level of awareness among schoolteachers and their acceptability is largely unknown despite their pivotal role in the overall upbringing of a female child. The existence of school health programmes therefore can be strengthened by including cervical cancer and HPV education as a major component of the programme, however, this does not exist in the country presently. This is supposed to help enrich the knowledge base of teachers on the existence of this preventable cancer. Furthermore, just as the level of awareness about cervical cancer vaccine varies according to population and profession, the level of acceptability of the vaccine varies [6-14].

It is important to note that the majority of studies done on the use of cervical cancer vaccines in Nigeria and Enugu in particular had dwelled more on health workers [5, 6]. The leadership and educating roles of secondary school teachers in the society will play a very important part in helping increase the knowledge base of cervical cancer and cervical cancer vaccines. It will equally play a role in accelerating the acceptance of the HPV vaccines among secondary school students, teachers, parents, friends, and even the general population. However, this population (secondary school teachers) concerning the above has not been fully studied. Female secondary school teachers are among the susceptible groups to cervical cancer, so they need the information. They are most times leaders in their women organizations and educate people generally. They also have relatives who are adolescents that are qualified to receive this vaccine. These female teachers are usually teaching young females who are in the appropriate age to receive these vaccines. Since they are females, they can easily have better standing to discuss these sexuality-related issues, especially among the female folks. Also, with a reduced number of male teachers in secondary schools, they are therefore the potential bedrock for the spread of information to the right population at the appropriate time about this vaccine as they do in other vaccines given to children. Finally, no meaningful progress can be made on the uptake of the cervical cancer vaccine if the level of awareness and acceptability of the vaccine remains low among the women population. Therefore, it is pertinent to assess the level of awareness and acceptability of the vaccine among female secondary school teachers as well as the factors influencing them.

## Methods

**Study area:** this study was carried out in secondary schools located in Enugu metropolis. Enugu metropolis is the capital of Enugu state. It has three local governments (Enugu South, Enugu North, and Enugu East).

**Study population:** the study population was female secondary school teachers in Enugu metropolis that were distributed in both public and private secondary schools in Enugu metropolis.

**Inclusion criteria:** all the female secondary school teachers in the selected secondary schools who gave their consent for this research were recruited for this study.

**Exclusion criteria:** all the female secondary school teachers who were not in the selected schools or that did not give consent for this study were excluded from this study. Also, teachers that were sick were excluded from the study.

**Study design and selection of participants:** this study was a cross-sectional analytical study involving female secondary school teachers in both public and private secondary schools in Enugu metropolis. A well-structured self-administered questionnaire was administered to consenting respondents from both the public and private secondary schools in Enugu metropolis. According to data from the Statistics Department of Enugu state Ministry of Education, there were 35 public secondary schools and 117 private secondary schools in Enugu metropolis. Also, there were 4608 female and 1577 male secondary school teachers in both public and private hospitals in the Enugu metropolis. Eighteen secondary schools were selected from the schools in three local government areas that made up the Enugu metropolis. Six secondary schools in all were selected from each local government using a multi-stage sampling technique. Out of these six, four public secondary schools randomly selected from the sample frame of all the public secondary schools in each local government. Also, two private secondary schools were selected from each local government purposefully and proportionately since there were wide variations in the number of female teachers in these private schools. Consecutively consenting female teachers were selected and interviewed from each school until the number of teachers allotted to that school was reached.

**Sample size estimation:** the minimum sample size (n) was determined by using the formula [15]:

n=Z2pqE2

Where Z was the coefficient of Z statistics obtained from the standard normal distribution table, p was the prevalence rate (in percent), q was 100 - p and E was the sampling error tolerated (in percent). Using an acceptability rate (p) of 95% for the HPV vaccine for a study carried out in Enugu, at a confidence limit of 95%, sampling error of 3%, and assuming a non-response rate of 5%, the calculated minimum sample size was 368 female secondary school teachers.

**Sampling method:** a multistage sampling technique was utilized in the selection of the sample used for this study. The three local governments in the metropolis were purposely selected. More public schools were chosen since they were more organized than the private schools in terms of knowing readily the staff strengths of the different schools. Simple random sampling was used to select the public secondary schools used while the private schools were purposely selected from each local government area (LGA). Four public secondary schools selected from each local government was done using the simple random method from the list of all the secondary schools in each local government in Enugu metropolis while the two private schools were selected purposefully from each LGA. The female secondary school teachers used were consecutively selected from each school until the number allotted for that school was reached. The questionnaire was then administered on the female secondary school teachers that gave consent for the research. Difficulties in understanding some questions were clarified by the interviewer. Data extracted from these questionnaires were used for analysis.

**Data analysis:** analysis was done with Special Package for Social Sciences (SPSS) version 20.0 Evaluation version software. The analysis was both descriptive and inferential with values set at 95% confidence level, a p-value of 0.05 was considered significant. Proportions were compared with Pearson´s Chi-square while means were compared with Student´s t-test. Data were presented using tables, graphs, charts, etc. as appropriate.

**Ethical considerations:** the ethical clearance certificate with number NHREC/05/01/2008B - FWA00002458 - IRB 00002323 for this research was obtained primarily from the Research and Ethics Committee of University of Nigeria Teaching Hospital Ituku/Ozalla, Enugu. Also, clearance was obtained from the state Post-Primary School Management (PPSMB) and principals of each of the schools used for this study.

## Results

The overall response rate was 94.25% (377/400). The mean age of the respondents was 37.46 years and most of the respondents were aged 30-39 years (40.6%). The majority of the respondents was spouses in their families (73.2%) and married in a monogamous family setting (75.9%). Most of the respondents had tertiary education (69.0%) and have taught for an average of 10.09 years with most having taught for 1-9 years (61.3%). Most of the teachers (75.3%) engaged in other forms of a job to bolster their economic status. The other details of the socio-demographic characteristics of the respondents are as shown in Table 1.

**Table 1:** the socio-demographic characteristics of the participants

Variables	Frequency (n=377)	Percent
**Age (Years)** 10-19	2	0.5
20-29	75	19.9
30-39	153	40.6
40-49	100	26.5
50-59	45	11.9
60-69	2	0.5
Mean=37.46 SD=9.15, Range=18-60		
**House-hold status**		
Head	37	9.8
Spouse	276	73.2
Children	64	17.0
**Number in the household category**		
1-5	193	51.2
6-10	176	46.7
11-15	8	2.1
**Years of experience category**		
1-5	157	41.6
6-10	95	25.2
11-15	38	10.1
16-20	46	12.2
21-25	19	5.0
26-30	13	3.4
31-35	9	2.4
Mean = 10.08, SD=8.072, Mode = 5, Range= 1-35		
**Level of Education**		
Secondary	17	4.5
Tertiary	260	69.0
Postgraduate	100	26.5
**Marital status**		
Married in monogamous	286	75.9
Married in polygamous	10	2.7
Divorced	2	0.5
Widowed	25	6.6
Single	52	13.8
Separated	2	0.5
**Teaching only**		
Yes	93	24.7
No	284	75.3

### Knowledge of cervical cancer vaccine among respondents

Less than half (41.9%) of the respondents had good knowledge of cervical cancer; only approximately 55% of the respondents have heard of cervical cancer vaccines while 48.3% of them knew that cervical cancer can be prevented through vaccination. The main sources of information were through healthcare workers (37.21%), mass media (30.23%), and friends (17.67). Other details are as shown in Table 2.

**Table 2:** the knowledge of cervical cancer vaccine among respondents

Variable	Category	Frequency (n=377)	Percent
Think cervical cancer can be prevented through vaccination	Yes	195	48.3
	No	182	51.7
Heard of cervical cancer vaccine?	Yes	208	55.2
	No	169	44.8
Knowledge of cervical cancer vaccination	Good knowledge	158	41.9
	Poor knowledge	218	57.8
**Sources of information**			
Mass media	Yes	65	30.23
	No	150	69.77
Friends	Yes	38	17.67
	No	177	82.33
Health workers	Yes	80	37.21
	No	135	62.79
Meetings	Yes	5	2.33
	No	210	97.67
Conference	Yes	24	11.16
	No	191	88.84
Schools	Yes	6	2.79
	No	209	97.21
Churches	Yes	11	5.12
	No	204	94.88
Social media	Yes	13	6.05
	No	202	93.95

### Acceptability of cervical cancer vaccine among the respondents

The majority (93.6%) of the respondents will recommend the cervical cancer vaccine to their children and students in their school if the vaccine was given free by the government and other bodies. The proportions of respondents who have taught their students about cervical cancer or HPV vaccines were only 14.6%. Only 9.0% of the teachers agreed they had a programme that addresses cervical cancer or cervical cancer vaccination. The majority of the respondents (85.4%) will recommend to their school to put in place programmes that address cervical cancer prevention especially administration cervical cancer vaccine to their students. The other levels of acceptance of the cervical cancer vaccine are as shown in Table 3.

**Table 3:** the levels of acceptability of cervical cancer vaccination by respondents

Variable	Responses	Frequency (n=377)	Percent
Respondents who have taught students cervical cancer	Yes	55	14.6
	No	322	85.4
Respondents who have taught students on cervical cancer vaccine	Yes	28	7.4
	No	349	92.6
Respondents with a programme addressing cervical cancer vaccination	Yes	34	9.0
	No	343	91.0
Respondents who will recommend to their school programmes addressing cervical cancer/vaccination	Yes	322	85.4
	No	27	7.4

### Uptake of cervical cancer vaccine by the respondents

Only 3.4% of the respondents have been vaccinated in the past while 5.6% of children were vaccinated by their mothers. Most (48.15%) of those who received the vaccination did not have any complication, while 22.2% reported pains at the injection and 11.11% reported fever after receiving the vaccine. The most common reasons for non-administration of the vaccines on eligible adolescents were non-availability (46.31%) and high cost of the HPV vaccines (25.12%). The details of the use of cervical cancer vaccines by the participants are shown in Table 4. Among respondents who vaccinated their children approximately 81% of them vaccinated females while the remaining 19% vaccinated both males and females. No male child was vaccinated alone.

**Table 4:** the use of cervical cancer vaccine by respondents on both themselves and their children, complications of vaccine use and reasons for non-administration

Variable	Category	Responses	Frequency (n=377)	Percent
**Use of cervical cancer vaccine**	Administration of vaccine by the respondent	Yes	13	3.4
		No	364	96.6
	Vaccinated children before	Yes	21	5.6
		No	356	94.4
		**Responses**	**Frequency (n=27)**	**Percent**
**Observed complications of vaccine administration**	fever	yes	3	11.11
		No	24	88.89
	convulsions	Yes	0	0
		No	27	100
	vomiting	Yes	0	0
		No	27	100
	Headache	Yes	2	7.41
		No	25	92.59
	Severe pains	Yes	6	22.22
		No	21	77.78
	Abscess formation	Yes	1	3.70
		No	26	96.30
	Redness at injection site	Yes	2	7.14
		No	25	92.59
	No complication	Yes	13	48.15
		No	14	51.85
		**Responses**	**Frequency (n=203)**	**Percent**
**Reasons for non-vaccination of children**	Fear of complication	Yes	16	7.88
		No	187	92.12
	The high cost of the vaccine	Yes	51	25.12
		No	152	74.88
	Promiscuity	Yes	1	0.49
		No	202	99.51
	Non-availability	Yes	94	46.31
		No	109	53.69
	Don´t believe in HPV vaccination	Yes	9	4.43
		No	194	95.56
	Poor information on the use of the vaccine	Yes	10	4.63
		No	193	95.07
	Unaware of the use of the vaccine	Yes	11	5.42
		No	192	94.58

### Factors associated with the administration of cervical cancer vaccines

Among the participants who have heard about the cervical cancer vaccine, there was a significant relationship between the age groups and vaccination of children against HPV infections. The extremes of ages were less likely to vaccinate their children with the cervical cancer vaccine (p = 0.001). The household status was also significantly associated with the administration of the vaccine. The respondents who were the head of their families probably because of the death of their husbands were less likely to give their children HPV vaccines (p = 0.002). Equally noted to be significantly consistent with the administration of HPV vaccine on adolescents were respondents having received the vaccines previously (p < 0.000), the existence of programmes in schools which addresses cervical cancer/ HPV vaccines (p < 0.001) and having previously taught student about cervical cancer/HPV vaccines (p = 0.025) (Table 5).

**Table 5:** factors associated with the administration of cervical cancer vaccines on children and relatives by respondents who have heard of the cervical cancer vaccine

Variable			Vaccinated children/relatives		Chi-square	P-value
Heard of cervical cancer vaccine?			Yes	No		
**Age group category**	Yes	10-19	1	1	19.804	0.001
		20-29	11	33		
		30-39	7	75		
		40-49	2	55		
		50-59	0	21		
		60-69	0	2		
**Marital status**	Yes	Married monogamous	13	140	8.397	0.078
		Married polygamous	0	8		
		Divorced	0	2		
		Widowed	0	9		
		Single	8	28		
**Years of teaching**	Yes	1-5	15	72	11.311	0.079
		6-10	3	46		
		11-15	3	23		
		16-20	0	27		
		21-25	0	10		
		26-30	0	6		
		31-35	0	3		
**Household status**	Yes	Head	0	14	12.225	0.002
		Spouse	11	142		
		Children	10	31		
**Number in the household**	Yes	1-5	10	85	0.371	0.831
		6-10	10	97		
		11-15	1	5		
**Level of education**	Yes	Secondary	2	8	1.580	0.454
		Tertiary	14	118		
		Post-graduate	5	61		
**Received cervical cancer vaccine**	yes	Vaccinated	10	3	68.225	0.000
		Not vaccinated	11	187		
**Any existing cervical cancer programme**	Yes	School programme exists	9	21	15.30	0.000
		No school programme	12	166		
**Taught students on the vaccine**	Yes	Have taught before	6	21		
		Have not taught students	15	166	5.026	0.025

### Economic factors associated with vaccination of children/relatives by respondents

Participant´s average monthly income was significantly associated with the administration of the HPV vaccines to their children. The more the monthly income the higher the likelihood of children being vaccinated by respondents (p=0.013) (Table 6).

**Table 6:** economic factors associated with vaccination of children/relatives by respondents

Variables			Vaccinated children/relatives		Chi-square	P-value
Heard of cervical cancer vaccine?			Yes	No		
Average monthly income category	Yes	<18000	1	9	12.704	0.013
		18000-49999	14	81		
		50000-99999	2	80		
		100000-199999	2	14		
		>199999	2	3		
Average Husband income	Yes	<18000	0	0	4.837	0.565
		18000-49999	0	23		
		50000-99999	6	56		
		100000-199999	6	50		
		200000-499999	1	18		
		500000-999999	1	4		
		> 999999	0	2		

### Predictors of administration of cervical cancer vaccines on children and relatives by respondents who have heard of cervical cancer vaccine

Women who received the cervical cancer vaccine in the past were about 6 times (AOR = 6.069; 95% CI; < 0.0001 - 0.041) more likely to vaccinate their children and relatives. No other variable was identified as a determinant of the administration of cervical cancer vaccines on children and relatives by respondents who have heard of the cervical cancer vaccine (Table 7).

**Table 7:** predictors of cervical cancer vaccination among respondents

Socio-demographic/other characteristics	Category	Adjusted odds ratio	p-value	95% Confidence interval	
				Lower Limit	Upper Limit
Age category	10-19 years	0.400	0.602	0.149	3.015
	70-79 years	1			
Household status	Others	0.565	0.551	0.089	3.641
	Head	1			
Marital status	Married monogamous	0.016	0.956	0.570	1.812
	Separated	1			
Received cervical cancer vaccine	Yes	6.069	**0.000**	0.000	0.041
	No	1			
Existing school program on cervical cancer	Yes	2.646	0.705	0.097	4.858
	No	1			
Teaches cervical cancer to students	Yes	1.464	0.444	0.088	2.907
	No	1			
Years of teaching	>30 years	1.170	0.054	0.987	5.601
	1 - 10years	1			
Average monthly income	<₦18,000	0.089	0.831	0.482	2.480
	₦1,000,000	1			
Non-food cost category	<₦50,000	0.348	0.547	0.227	2.193
	>₦2,000,000	1			

## Discussion

The proportion of women with good knowledge about the cervical cancer vaccine in this study was low (41.9%). This low level of correct information about the cervical cancer vaccine reflects the amount of information transmitted from teachers (respondents) to both the students and parents on the need for children to be vaccinated against HPV infections. This finding, therefore, suggests that a more concerted effort must be put towards educating teachers at all levels and other women groups alike on cervical cancer and the need for vaccination against it. This above finding was comparable with 39.1% recorded in a similar study [9] in Ibadan, Nigeria among women attending immunization centers. However, the finding of this study was higher than 19.7% that was recorded in a study [16] in Shomolu local government area, Lagos State Nigeria that studied mothers of adolescents in the study area. A similar study among women attending preventive care clinics in two rural communities in Honduras also recorded a low level of knowledge among the participants of the study where only 13% of the women studied were aware of the human papillomavirus vaccine [17]. The difference noted between these study populations may have been accounted for by the fact that the population involved in this study were teachers and were meant to have higher access to information of all sorts including health-related information. Likewise, in a study in Gwagwalada, Abuja northern Nigeria, the level of knowledge about the HPV vaccine was as low as 7.8% among antenatal attendees in a tertiary institution in that area [11]. The level of education and low level of medical care in that region of Nigeria may have played vital roles here.

However, the level of awareness in this study is quite lower than 62.7% that was recorded among health care workers in Enugu, Nigeria [6]. The difference that was observed may be because health care workers have more information and even recommend these vaccines to others and are therefore expected to have a piece of higher knowledge. The main sources of information about cervical cancer vaccine in this study were consistent with that in a similar study in Anambra state, Nigeria [18] where health workers (32.9%), mass media (television) (13.4%), friends (13.4%) and news media (14.6%) served as major sources of information on HPV infections and their implications. These veritable sources of information should be utilized in increasing the awareness of cervical cancer/cervical cancer vaccination among educated populations like teachers. Although knowledge was low among the respondents the proportion of the teachers that were willing to administer the vaccine to their adolescents was high (93.6%). This finding is consistent with that of similar studies in Honduras [17] and Ibadan, Nigeria [16], where the majority of the study participant mothers (91% and 88.9% respectively) despite their poor knowledge of cervical cancer vaccine were ready to recommend the HPV vaccines for their adolescents. Also, the acceptance level (91%) for HPV vaccines among healthcare workers in a healthcare institution in Enugu, Nigeria [6] is comparable to that recorded among teachers in this study. The extra education given during the interview session was quickly accepted by the respondents in this study, hence the high level of acceptance recorded among them. Other studies [19, 20] also recorded a high acceptance level of HPV vaccines among respondents.

The acceptance level was however higher than that of a study in Abakiliki, Ebonyi State, Nigeria where only 70% of the respondents (secondary school teachers) were willing to recommend the vaccine for their daughters [13]. Also, the acceptance level among mothers of adolescent girls in secondary schools in Bangkok, Thailand, and Eldoret, Kenya where only 76.9% and 60.3% respectively of the participants were ready to recommend HPV vaccines for their children [21, 22]. Among nurses in faith-based health care facilities in Cameroon, the acceptance level was 69.7% [23]. The slight difference noted here may have come from the extra education given by the interviewers in this study compared to the use of self-administration of questionnaires in these comparative studies. The role of education in increasing awareness and utilization of HPV vaccines among respondents was equally observed in a Lagos, Nigeria study [16]. Also, the level of education among teachers may have been responsible for the higher level of acceptance in this study compared to that of mothers used in the Bangkok study. The high level of acceptance among the respondents in this research shows that teachers can be utilized as a faster means of spreading information on cervical cancer and HPV vaccination. This is further supported by a high proportion of respondents (85.4%) who were willing to recommend their school programmes that will address cervical cancer or HPV vaccination. Similarly, in a study in Gwagwalada, Abuja Nigeria, 62.8% of the respondents (antenatal attendees) accepted to administer the HPV vaccines to their teenage girls [11]. This level acceptance is also in keeping with the general knowledge HPV vaccine which was poor in the same study. It was noted that only 3.4% of the respondents received the HPV vaccine, this proportion is lower than 20.7% that was recorded among immunization attendees in an immunization clinic in Ibadan, Nigeria [9]. The reason for the high proportion recorded in the Ibadan study may be because the center in the Ibadan study also doubles as an HPV vaccination center hence the large pool of mothers that was seen in that center. The proportion of children vaccinated (5.6%) by the respondents in this study was smaller than that of 49.2% that was recorded among healthcare workers in a study in Enugu, Nigeria [6]. The difference noted in these two studies may be accounted for by high awareness among the healthcare workers in the Enugu study and also the level of experience haboured by these healthcare workers compared to teachers that were the study population in this research.

The barriers to non-administration HPV vaccines to adolescents in this study include the high cost of the vaccine, non-availability, fear of side effects of the vaccine, and shortage of awareness of the vaccine. Similar barriers like high cost [6, 20, 24], unavailability/accessibility [6, 16], fear of side effects [12] etc. were also identified in similar studies. Other factors associated with non-vaccination as identified in this study include extremes of the age of mothers; mothers who were older and quite younger, vaccinated less of their children and relatives. The HPV vaccine is a new introduction to the health care industry, (especially in developing countries) it may not have to be available to these mothers to know about them even when they received antenatal care. The very young teachers may have to be so young to know much about cervical cancer hence the need for them to be thought when they are younger like during their secondary school ages. Therefore, any intervention towards increasing the uptake of cervical cancer vaccines by mothers should target this age group of mothers. Likewise, married status was identified as one of the associated factors with the vaccination of children. The support these mothers get from their husbands may have been instrumental. Importantly, having no prior knowledge of school programmes on cervical cancer vaccines, not receiving HPV vaccines by respondents in the past, and not teaching students previously about cervical cancer/HPV vaccine are major factors associated with non-vaccination of children. This finding suggests that teachers should be involved in the education of students and their parents about cervical cancer and cervical cancer vaccination since they guarantee vaccine uptake by adolescents. A previous history of vaccination of respondents was the only identified predictor of vaccination of the children and relatives of the respondents. This finding is similar to that obtained in a Zgorzelec, Poland study [25] and Canadian study [26] where the positive attitude of parents was identified as a predictor of parental willingness to vaccinate their children. Also, the past behaviour of mothers has equally been identified in an Italian study [27] as a predictor for mothers to vaccinate their children as noted in this study.

## Conclusion

The knowledge of secondary school teachers in Enugu metropolis on the cervical cancer vaccine was low and despite this, their acceptability for the vaccine was high. Also, only very few of the teachers have vaccinated their children citing high cost and unavailability of the vaccine as major reasons for non-administration of the vaccine to their children. The only predictor of vaccination of children was the mother’s previous history of HPV vaccination.

**Recommendations:** cervical cancer education programmes should be established in secondary schools (tertiary institutions inclusive) and among teachers since they have a high capacity to retain information and transfer the same to people around them. The government should also increase their efforts towards vaccinating mothers and mothers-to-be adolescents as this research has revealed that vaccinated mothers against HPV infections are more likely to vaccinate their children. Readjusting the current minimum wage upwards will help increase the economic status of women and this will equally increase uptake of the vaccine as noted in this study.

### What is known about this topic

- Vaccination of adolescents with human papilloma virus vaccines is a major preventive strategy for cervical cancer;
- Majority of studies done on the use of cervical cancer vaccines in Nigeria and Enugu in particular had dwelled more on health workers not other women groups;
- The secondary school teachers play roles in leadership and education of the society and will play be useful in helping increase the uptake of cervical cancer vaccine in the communities where they work.

### What this study adds

- The overall knowledge of the HPV vaccine was low but the acceptability was high among the female teachers who were aware of the vaccines;
- Only a small proportion of the female teachers have taught their students about cervical cancer or HPV vaccines and had a programme in their school that addresses cervical cancer or cervical cancer vaccination respectively;
- Previous vaccination of participants, existing school programmes addressing cervical cancer in schools of respondents, participants having taught students about cervical cancer and high economic status significantly increased the likelihood of participants vaccinating their adolescent daughters/relatives.
